# Y chromosome mosaicism is associated with age-related macular degeneration

**DOI:** 10.1038/s41431-018-0238-8

**Published:** 2018-08-29

**Authors:** Felix Grassmann, Christina Kiel, Anneke I. den Hollander, Daniel E. Weeks, Andrew Lotery, Valentina Cipriani, Bernhard H. F. Weber, on behalf of the International Age-related Macular Degeneration Genomics Consortium (IAMDGC)

**Affiliations:** 1 Institute of Human Genetics, University of Regensburg, Regensburg, Germany; 2 Department of Medical Epidemiology and Biostatistics, Karolinska Institutet, Stockholm, Sweden; 3 Department of Ophthalmology, Radboud University Medical Center, Nijmegen, The Netherlands; 4 Department of Human Genetics, Graduate School of Public Health, University of Pittsburgh, Pittsburgh, Pennsylvania USA; 5 Department of Biostatistics, Graduate School of Public Health, University of Pittsburgh, Pittsburgh, Pennsylvania USA; 6 Clinical and Experimental Sciences, Faculty of Medicine, University of Southampton, Southampton, UK; 7 University College London Institute of Ophthalmology, University College London, London, UK

**Keywords:** Predictive markers, Genome-wide association studies

## Abstract

Age-related macular degeneration (AMD) is the leading cause of blindness in industrialised countries, and thereby a major individual but also a socio-economic burden. Y chromosome loss in nucleated blood cells has been implicated in age-related diseases such as Alzheimer disease and was shown to be caused by increasing age, smoking and genetic factors. Mosaic loss of Y chromosome (mLOY) in peripheral blood was estimated from normalised dosages of genotyping chip data covering the male-specific region of the Y chromosome. After quality control, we assessed the association of mLOY on AMD risk in 5772 male cases and 6732 male controls. In controls the prevalence of mLOY increased significantly with age, which is consistent with previous reports. Importantly, mLOY was associated with late-stage AMD with genome-wide significance (OR: 1.332 [95% CI: 1.206; 1.472], *P* = 1.60e-08), independent of age, the AMD genetic risk score and the first two principle components of ancestry. Additionally conditioning on smoking behaviour had no influence on the observed association strength. mLOY was strongest associated in individuals aged between 65 and 75 years. Taken together, mLOY is significantly associated with risk for AMD, independent of known and potential confounding factors.

## Introduction

Age-related macular degeneration (AMD) is the most common cause of blindness in Western societies with age identified as the major risk factor for the disease [1]. The prevalence of AMD rises significantly with age; hence demographic changes towards an increased average life expectancy are anticipated to cause a growing individual and socioeconomic burden [2]. Apart from age, environmental/modifiable as well as genetic factors have firmly been established to be strongly associated with disease risk [1, 3, 4]. Previous findings revealed ambiguous results for an association of gender with AMD and its modulation by genetic variants [2, 5–7].

The latest genome-wide association study by the International AMD Genomics Consortium (IAMDGC) [8–10] has identified numerous regions in the genome involved in AMD pathogenesis. In total, at least 34 regions with currently more than 52 independent associated lead variants have been implicated in disease risk, explaining over 50% of the heritability and more than 30% of disease variance. Although the majority of the heritability can be explained by known genetic markers, no cellular pathway besides the complement cascade as part of the innate immune system was consistently found to be involved in AMD [11].

Mosaic loss of Y chromosome (mLOY) in peripheral blood is the most common acquired mutation in the process of normal aging in men, affecting about 1.8% of the genetic material in the human genome [12]. The prevalence of mLOY increases with age and can exceed 20% in male populations older than 80 years [13]. Furthermore, the occurrence of mLOY is strongly correlated with smoking behaviour [14]. Current smokers have a more than fourfold increased risk for mLOY [13], although this effect seems to be transient as smoking cessation can result in normal mLOY levels after several years [14, 15].

Recent reports suggested a role of Y chromosome loss in risk for all-cause mortality and common age-related disease such as cancer, Alzheimer disease as well as severe atherosclerosis [12–20]. Building on such reports, we aimed to evaluate the contribution of male Y chromosome mosaicism to the risk for late-stage AMD.

## Methods

### Description of dataset

Initially, data from 6529 males with late-stage AMD (NV, GA, or both, GA/NV AMD) and 7815 male control individuals without fundus changes associated with AMD, all probands unrelated and of European ancestry were provided by the IAMDGC [9]. Inclusion and exclusion criteria of the IAMDGC samples as well as detailed information on ophthalmological grading, quality control of genetic and phenotypic data as well as imputation are given in detail elsewhere [9]. The age of patients and controls reported by the individual studies was usually the date the blood was drawn and the diagnosis was assessed and/or confirmed in the recruitment centre or clinic [9]. For the present study, we excluded 1046 samples, since their DNA was not retrieved from whole blood. Smoking data were available for the samples from the Regensburg, EUGENDA (Nijmegen, the Netherlands and Cologne, Germany), UCLA/Pittsburgh, Southampton and Cambridge (UK) study groups, which are part of the IAMDGC dataset. In addition, smoking status was retrieved from dbGAP for the ARED study samples [21] (accession: phs000001.v3.p1). In total, we had smoking information for 2159 samples (ever smoked/never smoked), 1210 samples (current smoker status) and 956 samples (pack-years, Table 1). The study adhered to the tenets of the declaration of Helsinki and is HIPAA compliant.

**Table 1:** Baseline characteristics of the study population

	Cases	Controls	All	OR [95%CI]^a^	*P* ^a^
All ages [N/mLOY]	5772/1145	6732/918	12504/2063	1.332 [1.206; 1.472]	1.60 × 10^−08^
<65 years [N/mLOY]	538/69	1735/172	2273/241	1.316 [0.969; 1.771]	0.0738
65–70 years [N/mLOY]	675/108	1228/122	1903/230	1.748 [1.321; 2.311]	9.02 × 10^−05^
70–75 years [N/mLOY]	1131/209	1376/190	2507/399	1.422 [1.147; 1.764]	0.0013
75–80 years [N/mLOY]	1478/268	1191/175	2669/443	1.282 [1.042; 1.580]	0.0195
>80 years [N/mLOY]	1856/482	1161/255	3017/737	1.229 [1.034; 1.465]	0.0201
Ever smoked [%/*N*]	74.58/1137	63.6/1022	69.38/2159	1.688 [1.397; 2.041]	6.11 × 10^−08^
Current smoker [%/*N*]	16.57/519	8.39/691	11.9/1210	2.638 [1.804; 3.886]	6.92 × 10^−07^
Average pack-years (SD)	27.39 (26.2)	19.81 (23.88)	25.09 (25.75)	1.012 [1.006; 1.018]	2.47 × 10^−04^

*N* number of samples, *mLOY* number of samples with mLOY, *OR [95%]* odds ratio and 95% confidence interval of association between cases and controls (mLOY or smoking status), *P* *P* value of association between cases and controls, *SD* standard deviation

^a^Logistic regression models adjusted for age and the first two principal components of ancestry as well as the AMD genetic risk score

### Calculation of mLOY

All samples were genotyped on the same genotyping chip (Illumina HumanCoreExome—Goncalo 15038949). A continuous variable, the mean of the log R ratio (LRR), was used to estimate the degree of LOY for each subject and calculated as the mean LRR value of 608 SNP array probes that passed quality control (i.e. call rate >95% and not monomorphic) positioned within the male-specific region of the Y chromosome (mLRRY, hg19: 2,694,521 bp—59,034,049 bp) as described elsewhere [12, 15, 20]. To exclude samples with excessive noise, we computed the standard deviation (SD) of all LRR values of 30,789 SNP array probes on chromosome 1 that passed QC (mLRR1) and excluded 974 samples with a SD of mLRR1 greater than 0.28, as suggested by the manufacturer of the array. This allowed us to assess mLOY in a total of 12,504 individuals (Table 1). We defined males to have a significant level of mLOY in blood, if their mLRRY value was smaller than (1) the mean, (2) one or (3) two SDs from the mean mLRRY in controls. In subsequent analyses, we focused on mLOY individuals with mLRRY values smaller than one SD from the mean mLRRY in controls (2), since the fraction of mLOY individuals in controls was comparable to the frequency observed in other studies.

### Statistical analysis

We fit multivariable linear and logistic regression models to assess the association of mLRRY and mLOY with late-stage AMD, respectively, as implemented in R [22]. The analyses were adjusted for the first two principle components of ancestry, which were calculated from the imputed genotypes. In addition, where appropriate we accounted for the age at blood collection. To exclude a potential confounding effect of known and strong AMD associated variants, a genetic risk score (GRS) was computed for AMD with 52 independent variants from 34 loci, as previously reported [10, 23]. Briefly, we calculated the sum of risk increasing alleles from 52 AMD associated variants, weighted by the respective odds ratio of the variant. To account for smoking status in the sensitivity analysis, we considered individuals who ever smoked more than one pack-year as smokers and also adjusted for current smokers as well as the number of pack-years smoked, if available. We report the odds ratio of association and the slope of correlation as well as the respective 95% confidence intervals. The frequency of mLOY in cases and controls was plotted as a mosaicplot implemented in R and the mLRRY values across different age strata are shown as a boxplot, as implemented in R.

### Data availability

The genetic data of the IAMDGC can be accessed through dbGAP (accession: phs001039.v1.p1).

## Results

**Fig. 1:**
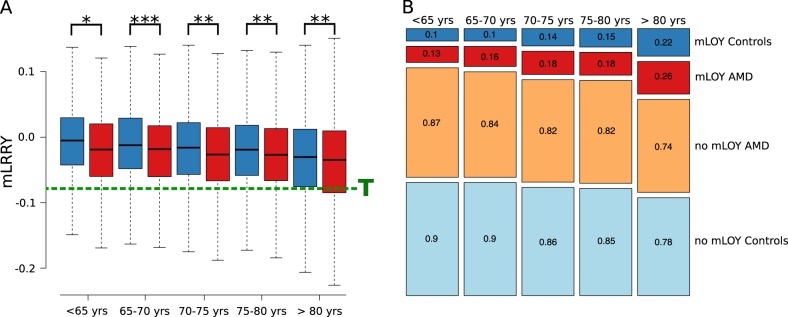
Boxplot and mosaicplot of mLOY in AMD patients and controls across different age groups. **a** The distribution of the mean LRR value across 608 SNP probes on the Y chromosome (mLRRY) for cases (red) and controls (blue) are depicted as a boxplot across different age strata. The threshold (T) for mLOY (mLRRY < −0.08) is depicted as a dashed green line. Significant differences between the mean mLRRY of cases and controls, as assessed by multivariate linear regression models adjusted for age, two principal components and AMD genetic risk score are depicted with asterisks: *P < 0.05; **P < 0.01; ***P < 0.001. **b** Patients with significant loss of Y chromosome (mLOY) were defined by a mLRRY smaller than one standard deviation from the mean mLRRY values of controls (mLRRY < −0.08). The occurrence of mLOY increased with age in controls (blue) as well as AMD patients (red). Conversely, cases (orange) as well as controls (light blue) with no mLOY were less frequently observed with increasing age. Absolute number of cases and the statistical assessment are given in Table 1

We investigated the association of mLOY in blood cells with late-stage AMD by using the currently largest collection of late-stage AMD patients and controls [9]. In total, 12,504 male individuals from 26 studies were included in the analysis (Table 1). All samples were genotyped simultaneously on the same chip in the same genotyping centre, resulting in little heterogeneity of mLOY across studies. Previous reports indicated a strong correlation between mLOY and age [12, 13]. As expected, the mean chromosomal dosage of the Y chromosome, as measured by the average LRR of all probes on the Y chromosome (mLRRY) decreased (*P* < 5.15 x 10^−64^) with age in AMD patients and AMD free controls (Fig. 1). We also found that current smokers (slope: −0.02 [95% CI: −0.03; 0.00], *P* < 0.05), as well as previous smokers (slope: −0.01 [95% CI: −0.02; 0.00], *P* < 0.05) had reduced levels of mLRRY. In addition, the number of pack-years smoked was inversely correlated to mLRRY (slope per ten pack-years: −0.002 [95% CI: 0.004; 0.000], *P* < 0.05).

Importantly, we observed a genome-wide significant association of mLRRY (OR per SD increase in mLRRY: 0.867 [95% CI: 0.833; 0.899], *P* = 8.75 × 10^−14^) with late-stage AMD, independent of age, ancestry and known AMD risk variants (summarised in a single GRS per person). Next, we defined mLOY as a binary variable in cases and controls by different agnostic mLRRY thresholds. We found that mLOY was statistically significant for association with AMD, either defined as (1) mLRRY smaller than the mean mLRRY in controls (OR: 1.249 [95% CI: 1.159; 1.345], *P* = 4.54 × 10^−09^), (2) mLRRY smaller than one SD from the mean of controls (OR: 1.332 [95% CI: 1.206; 1.472], *P* = 1.60 × 10^−08^) and (3) mLRRY smaller than two SDs from the mean of controls (OR: 1. 711 [95% CI: 1.406; 2.089], *P* = 1.03 × 10^−08^). The observed association was also independent of the above-mentioned confounders. In subsequent analyses, we focused on mLOY as defined by mLRRY smaller than one SD from the mean of mLRRY of controls (2), since this treshhold resulted in a similar fraction of mLOY in controls as previously described [15, 16].

When testing the association of mLOY with AMD in different age groups (Fig. 1), we observed the strongest association when restricting the analysis to individuals aged 65 to 75. The association strength (i.e. the odds ratio) decreased in older and younger individuals (Fig. 1). In addition, we investigated the effect of smoking status on the association strength with AMD. The observed association with late-stage AMD were not diminished when adjusting for ever smoking (OR per SD increase in mLRRY: 0.854 [95% CI: 0.762; 0.932]), current smoking status (OR per SD increase in mLRRY: 0.805 [95% CI: 0.701; 0.922]) or pack-years (OR per SD increase in mLRRY: 0.877 [95% CI: 0.735; 1.044]). Similarly, the association of mLOY did not decrease when adjusting for smoking status (data not shown).

## Discussion

Here, we report the association of mLOY with AMD risk, particularly in individuals between 65 and 75 years of age. We defined mLOY with agnostic thresholds from the mean and SD of the mLRRY values in controls, as previously published thresholds of mLRRY (such as −0.4 [13] or −0.15 [15]) resulted in fewer samples with mLOY than expected in this age range. This observation can be explained by the different genotyping chip architecture used in this study, which included a large exonic part to capture the variation of rare variants. However, our definition of mLOY based on one SD from the mean resulted in a similar fraction of mLOY in controls as expected in the respective age groups.

In line with independent results reported elsewhere [12, 13, 15], we also found that mLOY increased significantly with age in our study. Since age is the major risk factor for AMD, we adjusted the analysis by age, ensuring that the observed association is not driven by the increased age of our AMD samples compared to controls.

Previously, the strongest genetic associations for AMD were observed in or near genes of the complement system [9] and an increased complement activation has been shown to play a major role in AMD [11, 24, 25]. Enhanced and prolonged complement activity and thus immune activation might accelerate loss of Y chromosome and could be an important confounder in our analysis. However, the observed association of mLOY with AMD was independent of known AMD risk variants, demonstrated by adjusting the analysis for an AMD GRS, which revealed no influence on the observed association. Of note, genome-wide association studies aiming to uncover the genetic basis of mLOY have not reported any associated loci harbouring complement genes [20].

We observed that the association of mLOY with AMD is reduced in individuals over 75 years of age. This is in full agreement with a similar effect observed in the association of mLOY with Alzheimer disease [13], with decreased hazard ratios reported for older compared to younger individuals. It is known that in AMD genetic effects can be modulated by age [5, 7, 23]. It has been hypothesised that younger diseased individuals require more disease associated variants to explain the early onset of disease pathology. Similarly, mLOY at younger age may suggest an increased impairment and senescence of the immune system [12–14, 16, 17, 26], a process that is assumed to play a role in AMD and other immune related diseases [27, 28] as well as cancer [29, 30].

Taken together, we report a genome-wide significant association of mLOY with late-stage AMD independent of known and potential confounders. The mLOY marginally improved the AUC of the logistic regression model from 0.824 (GRS + smoking + age) to 0.827, which is consistent with the degree of added risk from other known, low penetrance genetic factors.

## Electronic supplementary material

Supplement Material IAMDGC Authors
