# Supplementary material for: Y chromosome mosaicism is associated with age-related macular degeneration
Source: Eur J Hum Genet. 2018 Aug 29;27(1):36–41. doi: 10.1038/s41431-018-0238-8 (PMC6303255; doi:10.1038/s41431-018-0238-8)
Supplement: Supplementary file 1 — Supplement Material IAMDGC Authors [file 41431_2018_238_MOESM1_ESM.docx]

**A large genome-wide association study of age-related macular degeneration highlights contributions of rare and common variants.**

*Nat Genet*. **48**:134-43, 2016

Authors:

Lars G Fritsche^1,100^, Wilmar Igl^2,100^, Jessica N Cooke Bailey^3,100^, Felix Grassmann^4,100^, Sebanti Sengupta^1,100^, Jennifer L Bragg-Gresham^1,5^, Kathryn P Burdon^6^, Scott J Hebbring^7^, Cindy Wen^8^, Mathias Gorski^2^, Ivana K Kim^9^, David Cho^10^, Donald Zack^11^–^15^, Eric Souied^16^, Hendrik P N Scholl^11,17^, Elisa Bala^18^, Kristine E Lee^19^, David J Hunter^20,21^, Rebecca J Sardell^22^, Paul Mitchell^23^, Joanna E Merriam^24^, Valentina Cipriani^25,26^, Joshua D Hoffman^27^, Tina Schick^28^, Yara T E Lechanteur^29^, Robyn H Guymer^30^, Matthew P Johnson^31^, Yingda Jiang^32^, Chloe M Stanton^33^, Gabriëlle H S Buitendijk^34,35^, Xiaowei Zhan^1,36,37^, Alan M Kwong^1^, Alexis Boleda^38^, Matthew Brooks^38^, Linn Gieser^38^, Rinki Ratnapriya^38^, Kari E Branham^39^, Johanna R Foerster^1^, John R Heckenlively^39^, Mohammad I Othman^39^, Brendan J Vote^6^, Helena Hai Liang^30^, Emmanuelle Souzeau^40^, Ian L McAllister^41^, Timothy Isaacs^41^, Janette Hall^40^, Stewart Lake^40^, David A Mackey^6,30,41^, Ian J Constable^41^, Jamie E Craig^40^, Terrie E Kitchner^7^, Zhenglin Yang^42,43^, Zhiguang Su^44^, Hongrong Luo^8^, Daniel Chen^8^, Hong Ouyang^8^, Ken Flagg^8^, Danni Lin^8^, Guanping Mao^8^, Henry Ferreyra^8^, Klaus Stark^2^, Claudia N von Strachwitz^45^, Armin Wolf^46^, Caroline Brandl^2,4,47^, Guenther Rudolph^46^, Matthias Olden^2^, Margaux A Morrison^48^, Denise J Morgan^48^, Matthew Schu^49^–^53^, Jeeyun Ahn^54^, Giuliana Silvestri^55^, Evangelia E Tsironi^56^, Kyu Hyung Park^57^, Lindsay A Farrer^49^–^53^, Anton Orlin^58^, Alexander Brucker^59^, Mingyao Li^60^, Christine A Curcio^61^, Saddek Mohand-Saïd^62^–^65^, José-Alain Sahel^25,62^–^67^, Isabelle Audo^62^–^64,68^, Mustapha Benchaboune^65^, Angela J Cree^69^, Christina A Rennie^70^, Srinivas V Goverdhan^69^, Michelle Grunin^71^, Shira Hagbi-Levi^71^, Peter Campochiaro^11,13^, Nicholas Katsanis^72^–^74^, Frank G Holz^17^, Frédéric Blond^62^–^64^, Hélène Blanché^75^, Jean-François Deleuze^75,76^, Robert P Igo Jr^3^, Barbara Truitt^3^, Neal S Peachey^18,77^, Stacy M Meuer^19^, Chelsea E Myers^19^, Emily L Moore^19^, Ronald Klein^19^, Michael A Hauser^78^–^80^, Eric A Postel^78^, Monique D Courtenay^22^, Stephen G Schwartz^81^, Jaclyn L Kovach^81^, William K Scott^22^, Gerald Liew^23^, Ava G Tan^23,^Bamini Gopinath^23^, John C Merriam^24^, R Theodore Smith^24,82^, Jane C Khan^41,83,84^, Humma Shahid^84,85^, Anthony T Moore^25,26,86^, J Allie McGrath^27^, Reneé Laux^3^, Milam A Brantley Jr^87^, Anita Agarwal^87^, Lebriz Ersoy^28^, Albert Caramoy^28^, Thomas Langmann^28^, Nicole T M Saksens^29^, Eiko K de Jong^29^, Carel B Hoyng^29^, Melinda S Cain^30^, Andrea J Richardson^30^, Tammy M Martin^88^, John Blangero^31^, Daniel E Weeks^32,89^, Bal Dhillon^90^, Cornelia M van Duijn^35^, Kimberly F Doheny^91^, Jane Romm^91^, Caroline C W Klaver^34,35^, Caroline Hayward^33^, Michael B Gorin^92,93^, Michael L Klein^88^, Paul N Baird^30^, Anneke I den Hollander^29,94^, Sascha Fauser^28^, John R W Yates^25,26,84^, Rando Allikmets^24,95^, Jie Jin Wang^23^, Debra A Schaumberg^20,96,97^, Barbara E K Klein^19^, Stephanie A Hagstrom^77^, Itay Chowers^71^, Andrew J Lotery^69^, Thierry Léveillard^62^–^64^, Kang Zhang^8,44^, Murray H Brilliant^7^, Alex W Hewitt^6,30,41^, Anand Swaroop^38^, Emily Y Chew^98^, Margaret A Pericak-Vance^22,101^, Margaret DeAngelis^48,101^, Dwight Stambolian^10,101^, Jonathan L Haines^3,99,101^, Sudha K Iyengar^3,101^, Bernhard H F Weber^4,101^, Gonçalo R Abecasis^1,101^ & Iris M Heid^2,101^

^1^Center for Statistical Genetics, Department of Biostatistics, University of Michigan, Ann Arbor, Michigan, USA. ^2^Department of Genetic Epidemiology, University of Regensburg, Regensburg, Germany. ^3^Department of Epidemiology and Biostatistics, Case Western Reserve University School of Medicine, Cleveland, Ohio, USA. ^4^Institute of Human Genetics, University of Regensburg, Regensburg, Germany. ^5^Kidney Epidemiology and Cost Center, Department of Internal Medicine–Nephrology, University of Michigan, Ann Arbor, Michigan, USA. ^6^School of Medicine, Menzies Research Institute Tasmania, University of Tasmania, Hobart, Tasmania, Australia. ^7^Center for Human Genetics, Marshfield Clinic Research Foundation, Marshfield, Wisconsin, USA. ^8^Department of Ophthalmology, University of California, San Diego and Veterans Affairs San Diego Health System, La Jolla, California, USA. ^9^Retina Service, Massachusetts Eye and Ear, Department of Ophthalmology, Harvard Medical School, Boston, Massachusetts, USA. ^10^Department of Ophthalmology, Perelman School of Medicine, University of Pennsylvania, Philadelphia, Pennsylvania, USA. ^11^Department of Ophthalmology, Wilmer Eye Institute, Johns Hopkins University School of Medicine, Baltimore, Maryland, USA. ^12^Department of Molecular Biology and Genetics, Johns Hopkins University School of Medicine, Baltimore, Maryland, USA. ^13^Department of Neuroscience, Johns Hopkins University School of Medicine, Baltimore, Maryland, USA. ^14^Institute of Genetic Medicine, Johns Hopkins University School of Medicine, Baltimore, Maryland, USA. ^15^Institue de la Vision, Université Pierre et Marie Curie, Paris, France. ^16^Hôpital Intercommunal de Créteil, Hôpital Henri Mondor, Université Paris Est Créteil, Créteil, France. ^17^Department of Ophthalmology, University of Bonn, Bonn, Germany. ^18^Louis Stokes Cleveland Veterans Affairs Medical Center, Cleveland, Ohio, USA. ^19^Department of Ophthalmology and Visual Sciences, University of Wisconsin, Madison, Wisconsin, USA. ^20^Department of Epidemiology, Harvard School of Public Health, Boston, Massachusetts, USA. ^21^Department of Nutrition, Harvard School of Public Health, Boston, Massachusetts, USA. ^22^John P. Hussman Institute for Human Genomics, Miller School of Medicine, University of Miami, Miami, Florida, USA. ^23^Centre for Vision Research, Department of Ophthalmology and Westmead Millennium Institute for Medical Research, University of Sydney, Sydney, New South Wales, Australia. ^24^Department of Ophthalmology, Columbia University, New York, New York, USA. ^25^University College London Institute of Ophthalmology, University College London, London, UK. ^26^Moorfields Eye Hospital, London, UK. ^27^Center for Human Genetics Research, Vanderbilt University Medical Center, Nashville, Tennessee, USA. ^28^Department of Ophthalmology, University Hospital of Cologne, Cologne, Germany. ^29^Department of Ophthalmology, Radboud University Medical Centre, Nijmegen, the Netherlands. ^30^Centre for Eye Research Australia, University of Melbourne, Royal Victorian Eye and Ear Hospital, East Melbourne, Victoria, Australia. ^31^South Texas Diabetes and Obesity Institute, School of Medicine, University of Texas Rio Grande Valley, Brownsville, Texas, USA. ^32^Department of Biostatistics, Graduate School of Public Health, University of Pittsburgh, Pittsburgh, Pennsylvania, USA. ^33^Medical Research Council (MRC) Human Genetics Unit, Institute of Genetics and Molecular Medicine, University of Edinburgh, Edinburgh, UK. ^34^Department of Ophthalmology, Erasmus Medical Center, Rotterdam, the Netherlands. ^35^Department of Epidemiology, Erasmus Medical Center, Rotterdam, the Netherlands. ^36^Quantitative Biomedical Research Center, Department of Clinical Science, University of Texas Southwestern Medical Center, Dallas, Texas, USA. ^37^Center for the Genetics of Host Defense, University of Texas Southwestern Medical Center, Dallas, Texas, USA. ^38^Neurobiology, Neurodegeneration and Repair Laboratory (N-NRL), National Eye Institute, US National Institutes of Health, Bethesda, Maryland, USA. ^39^Department of Ophthalmology and Visual Sciences, University of Michigan, Kellogg Eye Center, Ann Arbor, Michigan, USA. ^40^Department of Ophthalmology, Flinders Medical Centre, Flinders University, Adelaide, South Australia, Australia. ^41^Centre for Ophthalmology and Visual Science, Lions Eye Institute, University of Western Australia, Perth, Western Australia, Australia. ^42^Sichuan Provincial Key Laboratory for Human Disease Gene Study, Hospital of the University of Electronic Science and Technology of China and Sichuan Provincial People’s Hospital, Chengdu, China. ^43^Sichuan Translational Medicine Hospital, Chinese Academy of Sciences, Chengdu, China. ^44^Molecular Medicine Research Center, State Key Laboratory of Biotherapy, West China Hospital, Sichuan University, Chengdu, China. ^45^EyeCentre Southwest, Stuttgart, Germany. ^46^University Eye Clinic, Ludwig Maximilians University, Munich, Germany. ^47^Department of Ophthalmology, University Hospital Regensburg, Regensburg, Germany. ^48^Department of Ophthalmology and Visual Sciences, University of Utah, Salt Lake City, Utah, USA. ^49^Department of Medicine (Biomedical Genetics), Boston University Schools of Medicine and Public Health, Boston, Massachusetts, USA. ^50^Department of Ophthalmology, Boston University Schools of Medicine and Public Health, Boston, Massachusetts, USA. ^51^Department of Neurology, Boston University Schools of Medicine and Public Health, Boston, Massachusetts, USA. ^52^Department of Epidemiology, Boston University Schools of Medicine and Public Health, Boston, Massachusetts, USA. ^53^Department of Biostatistics, Boston University Schools of Medicine and Public Health, Boston, Massachusetts, USA. ^54^Department of Ophthalmology, Seoul Metropolitan Government Seoul National University Boramae Medical Center, Seoul, Republic of Korea. ^55^Centre for Experimental Medicine, Queen’s University, Belfast, UK. ^56^Department of Ophthalmology, University of Thessaly, School of Medicine, Larissa, Greece. ^57^Department of Ophthalmology, Seoul National University Bundang Hospital, Seongnam, Republic of Korea. ^58^Department of Ophthalmology, Weill Cornell Medical College, New York, New York, USA. ^59^Scheie Eye Institute, Department of Ophthalmology, University of Pennsylvania Perelman School of Medicine, Philadelphia, Pennsylvania, USA. ^60^Department of Biostatistics and Epidemiology, University of Pennsylvania Perelman School of Medicine, Philadelphia, Pennsylvania, USA. ^61^Department of Ophthalmology, University of Alabama at Birmingham, Birmingham, Alabama, USA. ^62^INSERM, Paris, France. ^63^Institut de la Vision, Department of Genetics, Paris, France. ^64^Centre National de la Recherche Scientifique (CNRS), Paris, France. ^65^Centre Hospitalier National d’Ophtalmologie des Quinze-Vingts, Paris, France. ^66^Fondation Ophtalmologique Adolphe de Rothschild, Paris, France. ^67^Académie des Sciences–Institut de France, Paris, France. ^68^Department of Molecular Genetics, Institute of Ophthalmology, London, UK. ^69^Clinical and Experimental Sciences, Faculty of Medicine, University of Southampton, Southampton, UK. ^70^University Hospital Southampton, Southampton, UK. ^71^Department of Ophthalmology, Hadassah Hebrew University Medical Center, Jerusalem, Israel. ^72^Center for Human Disease Modeling, Duke University, Durham, North Carolina, USA. ^73^Department of Cell Biology, Duke University, Durham, North Carolina, USA. ^74^Department of Pediatrics, Duke University, Durham, North Carolina, USA. ^75^Centre d’Etude du Polymorphisme Humain (CEPH) Fondation Jean Dausset, Paris, France. ^76^Commissariat à l’Energie Atomique et aux Energies Alternatives (CEA), Institut de Génomique, Centre National de Génotypage, Evry, France. ^77^Cole Eye Institute, Cleveland Clinic, Cleveland, Ohio, USA. ^78^Department of Ophthalmology, Duke University Medical Center, Durham, North Carolina, USA. ^79^Department of Medicine, Duke University Medical Center, Durham, North Carolina, USA. ^80^Duke Molecular Physiology Institute, Duke University Medical Center, Durham, North Carolina, USA. ^81^Bascom Palmer Eye Institute, University of Miami Miller School of Medicine, Naples, Florida, USA. ^82^Department of Ophthalmology, New York University School of Medicine, New York, New York, USA. ^83^Department of Ophthalmology, Royal Perth Hospital, Perth, Western Australia, Australia. ^84^Department of Medical Genetics, Cambridge Institute for Medical Research, University of Cambridge, Cambridge, UK. ^85^Department of Ophthalmology, Cambridge University Hospitals National Health Service (NHS) Foundation Trust, Cambridge, UK. ^86^Department of Ophthalmology, University of California San Francisco Medical School, San Francisco, California, USA. ^87^Department of Ophthalmology and Visual Sciences, Vanderbilt University, Nashville, Tennessee, USA. ^88^Casey Eye Institute, Oregon Health and Science University, Portland, Oregon, USA. ^89^Department of Human Genetics, Graduate School of Public Health, University of Pittsburgh, Pittsburgh, Pennsylvania, USA. ^90^School of Clinical Sciences, University of Edinburgh, Edinburgh, UK. ^91^Center for Inherited Disease Research (CIDR) Institute of Genetic Medicine, Johns Hopkins University School of Medicine, Baltimore, Maryland, USA. ^92^Department of Ophthalmology, David Geffen School of Medicine, Stein Eye Institute, University of California, Los Angeles, Los Angeles, California, USA. ^93^Department of Human Genetics, David Geffen School of Medicine, University of California, Los Angeles, Los Angeles, California, USA. ^94^Department of Human Genetics, Radboud University Medical Centre, Nijmegen, the Netherlands. ^95^Department of Pathology and Cell Biology, Columbia University, New York, New York, USA. ^96^Center for Translational Medicine, Moran Eye Center, University of Utah School of Medicine, Salt Lake City, Utah, USA. ^97^Division of Preventive Medicine, Brigham and Women’s Hospital, Harvard Medical School, Boston, Massachusetts, USA. ^98^Division of Epidemiology and Clinical Applications, Clinical Trials Branch, National Eye Institute, US National Institutes of Health, Bethesda, Maryland, USA. ^99^Institute for Computational Biology, Case Western Reserve University School of Medicine, Cleveland, Ohio, USA.

^100^These authors contributed equally to this work. ^101^These authors jointly supervised this work.
